# Effect of a 6-Week Structured Exercise Intervention on TNF-α During the Chronic Recovery Phase of a Burn Injury Compared to Regular Daily Activities: A Pilot Randomised Cross-Over Trial

**DOI:** 10.3390/ebj7010011

**Published:** 2026-02-12

**Authors:** Tyler Jerome Osborne, Grant Rowe, Dale W. Edgar, Mark Fear, Fiona M. Wood, Timothy Fairchild, Brook Galna, Pippa Kenworthy, Brad Wall

**Affiliations:** 1 School of Allied Health [Exercise Science], Murdoch University, Perth, WA 6150, Australia; t.fairchild@murdoch.edu.au (T.F.);; 2 Centre for Healthy Ageing, Health Futures Institute, Murdoch University, Perth, WA 6150, Australia; 3 Fiona Wood Foundation, Fiona Stanley Hospital, Perth, WA 6150, Australiadale.edgar@health.wa.gov.au (D.W.E.);; 4 School of Medical and Health Sciences, Edith Cowan University, Perth, WA 6027, Australia; 5 School of Psychology, College of Health and Education, Murdoch University, Perth, WA 6150, Australia; 6 Burns Service of Western Australia, Fiona Stanley Hospital, South Metropolitan Health Service, Perth, WA 6150, Australia; 7 Institute for Health Research, The University of Notre Dame Australia, Fremantle, WA 6160, Australia; 8 Burn Injury Research Unit, University of Western Australia, Perth, WA 6009, Australia; 9 Translational and Clinical Research Unit, Newcastle University, Newcastle upon Tyne NE1 7RU, UK

**Keywords:** burns, inflammation, metabolism, exercise, rehabilitation

## Abstract

Background: Burn injury induces a prolonged inflammatory response that may contribute to long-term metabolic dysfunction. Exercise is known to reduce inflammation in various clinical populations; however, its effect on chronic post-burn inflammation remains unclear. This crossover trial investigated the impact of a 6-week exercise intervention on tumour necrosis factor-alpha (TNF-α) in adults with non-severe burns sustained more than one year prior. Methods: Twenty-one participants were randomised to complete either a 6-week exercise program or a control period first, separated by a 4-week washout. The exercise program comprised three supervised sessions per week of combined resistance and cardiovascular training. Primary (TNF-α) and secondary (muscle strength, cardiovascular fitness) outcomes were assessed pre- and post-intervention. Results: Fifteen participants completed the protocol with high adherence (90.4%). Exercise significantly improved quadriceps strength and cardiovascular fitness, confirming the intervention’s safety and efficacy in this cohort. However, TNF-α concentrations were not elevated at baseline and did not significantly change following exercise compared with control (mean difference: +0.5 pg·mL^−1^, *p* = 0.249). Exercise is safe and beneficial for non-severely burned patients who sustained their injury > 1 year ago. However, inflammation was not elevated in this cohort, precluding our ability to test the effects of exercise on chronic inflammation.

## 1. Introduction

A burn injury is traumatic and can have long-lasting deleterious effects on the immune, metabolic, cardiovascular and musculoskeletal systems [1]. The physiological impact of a burn extends far beyond the initial injury [2,3,4,5], with long term consequences of increased inflammation being observed post-burn [3,6]. Despite non-severe burns (≤20% TBSA (total body surface area)) making up the majority of admitted burn cases in Australia [7], it remains unclear what the best strategy to mitigate these long-term inflammatory elevations is. Therefore, there is a current need to identify and develop long-term treatment strategies to mitigate the inflammatory response in patients with non-severe burns.

Currently, burn injuries may be treated during acute hospitalisation with medications, such as propranolol to induce negative chronotropy and attenuate hypermetabolism [2], while anti-microbials are used to treat infections [8]. However, these treatments have notable side effects including weight gain, cardiac arrythmias and hyperglycaemia, and their long-term impacts are unknown [9]. In contrast, exercise is a common treatment that has been found to be safe in the burn population and improve lean body mass and skeletal muscle synthesis [10], as well as improve other clinical and functional outcomes in the acute post-burn period [11,12,13]. Despite evidence across diverse clinical populations that exercise reduces inflammatory activity and improves function, it remains rarely prescribed long after burn injury [10,14]. Whilst exercise in the post-acute recovery phase has not yet been explored in non-severe burns, our team’s acute phase trial (>5% TBSA) compared exercise with control and tracked C-reactive protein (CRP) over time [15,16]. It was found that higher CRP at 26 weeks post-burn was significantly associated with poorer physical quality of life domains (β = −0.02 to −0.51, *p* < 0.001) [16]. Additionally, participants in the exercise intervention group had a significantly faster reduction in CRP as a result of the exercise (mean difference: −100 pg·mL^−1^, 95% confidence intervals: 50 to 170 pg·mL^−1^, *p* < 0.002) [16]. These findings indicate that exercise may have a meaningful role in moderating inflammation during recovery, offering potential benefits for long-term health and quality of life after burn injury.

One of the immediate physiological responses to a burn injury is a substantial release of both pro-inflammatory [tumour necrosis factor-α (TNF-α), and interleukins (IL) 6, 8] and anti-inflammatory markers (IL-2, 4, 10 and granulocyte-colony stimulating factor). However, the exact time course and magnitude of this response is unclear [2,4,17,18,19,20,21]. While the concentration of inflammatory markers after severe burn injury has been regularly reported as remaining elevated for up to 60 days post-burn [2], emerging evidence also suggests that some inflammatory markers remain abnormal post-burn—potentially exceeding 3 years [6]. For example, multiple studies show active hypertrophic scar changes, a type of scarring characterised by chronic inflammation, in the years and decades post-burn injury [6,22,23,24,25,26]. Effective strategies are required to mitigate the long-lasting inflammatory response to burns.

Systemic burden persists long after a burn. In the first five years post-injury, patients show higher rates of age-related disease–including cancer, diabetes and cardiovascular disease [5,27,28]. Consistent with this elevated risk, adults with a prior burn had 2.2-fold higher hospital admissions for type 2 diabetes than uninjured peers [29], 1.5-fold higher cardiovascular-related admission rates, and—once admitted—a 2.9-fold longer length of stay [30]. Moreover, various types of cancers occur more frequently in patients who have sustained a burn, compared to an uninjured cohort, across all ages, cohorts and sexes, including cancer of the buccal cavity, larynx, liver, respiratory tract, breast and genitals [31]. However, this could be due to a higher incidence of adverse health-related behaviours and pre-existing health conditions [32]. Excessive cytokine release [20], hyperglycaemia and increased energy expenditure [3], and muscle wastage [33] have been proposed as mechanisms explaining the increased risk of these diseases [29,30,31,34]. The latent risk for these diseases after burn injury warrants further investigation into: (i) understanding the long-term physiological changes post-burn and (ii) developing effective treatment strategies to minimise negative long-term outcomes. As research priorities, these are strongly supported by consumers around the world [35].

Exercise during the acute and subacute post-burn phases is feasible and safe, and it improves lean body mass and skeletal muscle protein synthesis, among other outcomes [36,37]. In summary, these benefits support exercise as an accessible, low-cost, low-risk therapy with the potential to reduce longer-term physiological consequences of burn injury.

The aims of this randomised crossover trial were

(i) To determine if 6 weeks of structured exercise was safe, well-adhered to and beneficial for patients who sustained a non-severe burn > 1 year ago.
(ii) To determine if burn-induced inflammation is elevated above human norms in patients who sustained a non-severe burn > 1 year ago.
(iii) To investigate the effects of a 6-week exercise intervention compared to standard daily activities on burn-induced inflammation in patients who sustained their non-severe burn > 1 year ago.

## 2. Materials and Methods

### 2.1. Participants

Participants were recruited from the State Adult Burns Service at Fiona Stanley Hospital between September 2020 and July 2021. Participants were considered for inclusion in the study if they had sustained a ≤20% TBSA burn injury over 1 year ago and were over 18 years of age. Potential participants were excluded if they had an acquired or pre-existing neurological injury or disease which influence the capacity to complete exercise (e.g., nerve injury, multi-trauma, spinal cord injury, CVA/CNS lesions), had unstable cardiac conditions, were non-English speaking, or had a surgical intervention within the study period.

The study was approved by the South Metropolitan Health Service Humans Research Ethics Committee [RGS3381] and Murdoch University Human Research Ethics Committee [2020/501] and registered on the Australian and New Zealand Clinical Trials Registry [ACTRN12620000237987p].

### 2.2. Procedure

**Figure 1:**
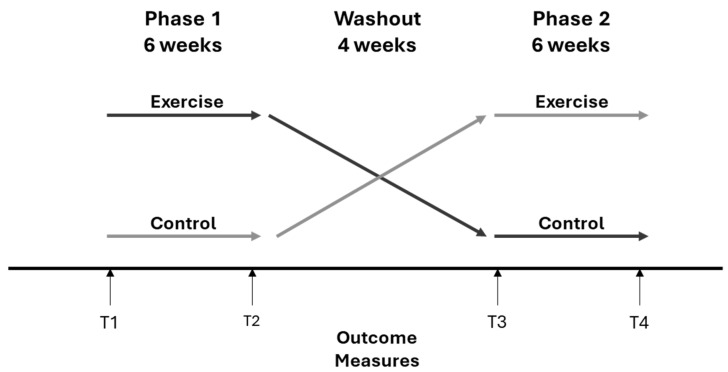
Study design illustrating the crossover design and 16-week study period [37]. Note: T1 = time point 1 [baseline of phase 1], T2 = time point 2 [end of phase 1], T3 = time point 3 [baseline of phase 2], and T4 = time point 4 [end of phase 2].

Within the crossover design, participants were randomised at recruitment to one of two sequences: either the control arm followed by the exercise arm [CON-EX], or the exercise arm followed by the control arm [EX-CON], with the phases separated by a 4-week washout [16] (Figure 1). The exercise arm included both resistance and aerobic training. In the control arm, participants were asked to continue with their usual daily activity and avoid strength training.

### 2.3. Exercise Condition

Due to the COVID-19 pandemic, restrictions were placed on participants attending the hospital for exercise sessions. To overcome this, participants were screened via the Anaesthetic Perioperative Clinic (APOC) Patient Health questionnaire, and those who were assessed as ‘low risk of adverse events’ were presented with two exercise intervention options: (1) supervised training at the hospital or (2) remote training on their own, with exercise support, prescription and monitoring through the diarising available on the PhysiApp smartphone app (Physitrack Version 4.074 Limited, London, UK). Those assessed as moderate or high risk of adverse events to exercise were required to attend onsite for supervised training at the hospital. In both supervised and remote training, the exercise was prescribed (and supervised in the case of in-hospital exercise) by an Accredited Exercise Physiologist, Exercise Scientist, or Physiotherapist.

The exercise intervention consisted of three training sessions per week, for six weeks. Participants during this period were also encouraged to complete 20 min of moderate intensity walking on non-training days; however, this was not recorded. The training sessions included aerobic and resistance training of moderate-to-vigorous activity for 60 min. Resistance training consisted of three sets of 8–12 repetitions of six multi-joint strength exercises: squats, leg press, hip bridges, chest press, shoulder press and seated row variations, completed at a rating of perceived exertion (RPE) of six to eight out of ten [38]. Previous reports indicate that performing near fatiguing sets of 10 repetitions is equivalent to approximately lifting a 70% one repetition-maximum (1-RM) load to near failure [39]. Twenty minutes, completed as two 10 min blocks of aerobic activity at a RPE of six to eight was also completed [37]. The total distance covered during aerobic activity for the session was recorded. Participants who trained remotely differed only with the exercises prescribed customised to equipment available to them, with equivalent training intensity, duration and frequency of those whose training was supervised. For example, the remote participants performed either running, cycling and body weight movements such as star jumps and mountain climbers for their aerobic training.

### 2.4. Control Condition

As noted, all participants were asked to also complete the control condition. During the control period, participants were asked to continue with their normal daily activities, including exercise for leisure and recreation, but to avoid deliberate strength training. For the duration of the control condition, their activity was recorded at each assessment using the IPAQ long-format questionnaire. The crossover nature of this study meant participants completed both exercise and control interventions.

### 2.5. Outcome Measures

A comprehensive outcome battery was completed throughout the study. Outcome measures were collected a total of four times (T1–T4), at baseline (T1, T3) and at completion of each study period (T2, T4), except for metabolic flexibility which was only collected before and after the exercise intervention, and HbA1c which was only collected at T1 (Figure 1).

#### 2.5.1. Primary Outcome

##### Cytokine TNF-α Levels

TNF-α was assessed as a marker of systemic inflammation. An elevated concentration of TNF-α is indicative of pro-inflammatory activity, and there is evidence suggesting exercise can reduce inflammation [1,4,5]. Blood samples were collected by venepuncture into 4 mL lithium heparin blood collection tubes, before and at the end of both the exercise and control phase, to determine the state of systemic inflammation after burn. Blood samples were centrifuged, and the plasma was stored at −80 °C for batch analysis of TNF-α by multiplexed immunosorbent assay (Thermofisher ProcartaPlex, Waltham, MA, USA). Analysis of TNF-α using these methods has been reported as a reliable measure with a 14-day re-test reliability 95% limits of agreement: −17 to 10 pg·mL^−1^ [40].

#### 2.5.2. Secondary Outcomes

##### Metabolic Flexibility

Expired air, fractional O_2_ and CO_2_ were collected via indirect calorimetry, using a single-use mouthpiece attachment, to determine $\dot{V}$O_2_ and $\dot{V}$CO_2_ using a metabolic cart. This was only performed before and after the exercise intervention to reduce burden on the participant (Quark RMR, COSMED, Rome, Italy) [41]. The approach to measuring metabolic flexibility is to assess baseline, maximum and rate of change in RER in response to glucose consumption after fasting [42]. In this case, a faster and larger increase in RER indicates better metabolic flexibility, whereas slower and smaller increases in RER are observed in hyperglycaemic populations such as people with type 2 diabetes.

Relative contributions of fat and carbohydrate metabolism were estimated from the resting RER measured using indirect calorimetry and calculated from $\dot{V}$O_2_ and $\dot{V}$CO_2_ as described in Equation (1) [43]:

(1) $$\mathrm{Respiratory}\mathrm{exchange}\mathrm{ratio}=\dot{V}{\mathrm{CO}}_{2}÷\dot{V}O_{2}$$

where $\dot{V}$CO_2_ = Flow rate [mL·min^−1^] of carbon dioxide produced and $\dot{V}$O_2_ = Flow rate [mL·min^−1^] of oxygen consumed RER approaching 1.0 represents predominant glucose metabolism, while RER = 0.7 represents predominant fatty acid oxidation [44].

Participants consumed a drink containing 75 g of glucose. The post-meal metabolic assessment lasted for 60 min to assess the response of fuel switching after the nutritional stimulus of the standardised meal and followed the Compher et al. guidelines outlined previously [45].

Due to COVID-19 restrictions and the need to minimise the spread of any potential viruses by participants using the equipment, a snorkel-like single use mouthpiece was used, also allowing patients with facial burns to be included, so long as they were able to form a seal around the mouthpiece.

##### Inflammatory Markers

A fresh whole blood sample to assess HbA1c was collected in ethylenediaminetetraacetic acid [EDTA] tubes by venepuncture. Inflammatory markers interleukins 2 and 6 were collected as blood samples in EDTA tubes by venepuncture and separated into plasma fraction by centrifugation at 1300 RCF [relative centrifugal force] for 10 min. The plasma was then collected via pipet and stored at −80 °C until analysis of inflammatory markers. The analysis of inflammatory markers has been found to be a reliable measure, with 14-day re-test reliability 95% limits of agreement: HbA1c: −0.38 to 0.92%, IL-2: −38.88 to 22.49 pg∙mL^−1^, IL-6: −12.68 to 9.03 pg∙mL^−1^ [40,46].

##### Muscle Strength

Muscle strength was measured before and after the exercise and control phases, using handheld dynamometry as per a previously established clinical protocol, recorded in kilograms [47]. The outcomes assessed for the upper and lower limb were grip strength, isometric biceps, and quadriceps strength.

To minimise the impact of familiarisation, the first effort was discarded and only data from the second and third attempts were averaged for analysis [47]. Using data from the second and third assessments of maximum voluntary isometric contraction, a mean strength value was generated for combined left- and right-sided elbow flexion, knee extension and grip strength.

##### Cardiorespiratory Fitness

Cardiorespiratory fitness was measured using the sub-maximal modified Chester Step Test (mCST) [48]. The test required participants to step on and off a 20 cm step at a standardised cadence that increase every 2 min. For the first 2 min the cadence was 15 steps·min^−1^, and the cadence increased by 5 steps·min^−1^ at the end of every second minute to a maximum cadence of 35 steps·min^−1^. The criteria for test completion were either (1) attainment of a heart rate equal to 80% of their age-predicted maximum or (2) the onset of intolerable symptoms of breathlessness or leg fatigue [48]. The mCST was completed on all participants after their blood was drawn to minimise the effect the short burst of exercise may have on inflammatory markers.

##### Body Composition

Bioimpedance spectroscopy (BIS) was used to evaluate estimates of individuals body composition. Individuals were asked to rest supine, and electrodes were placed on the right hand and foot as per manufacturers guidelines for a tetrapolar electrode arrangement [49]. The SFB7 (Impedimed, Pinkenba Queensland, Australia) was used and whole-body BIS measures were taken three times, with one-second intervals between measurements [49]. Bioimpedance estimates total body water through the resistance of the body to a small alternating current [50]. Intracellular resistance refers to the opposition a low-frequency electrical current encounters as it passes through intracellular fluid. Lean mass is calculated using the intracellular resistance, along with sex and age, and fat mass is then interpreted as the difference between total body mass and lean mass [50]. The use of bioimpedance to measure body composition has been validated in the general population and some clinical populations with gold standard measures including DEXA and MRI [51,52]. Bioimpedance has been demonstrated to be reliable and valid for measuring compartment volumes in acute burn injury (ICC = 0.998, 95% CI: 0.996 to 0.999) [53,54,55]; however, its use to estimate body composition has not yet been validated as an accurate method of assessment in burns patients.

### 2.6. Statistical Analysis

Data collected within this study was analysed using SPSS (version 29), and statistical significance was set at *p* ≤ 0.05. Descriptive statistics are presented as frequencies and percentages for categorical variables and mean and standard deviation for continuous variables, and all descriptors were normally distributed as determined by Shapiro–Wilk test (*p* > 0.05). The residuals were tested for normality and sphericity to meet the assumptions for repeated measures ANOVA.

To address the primary aim of this study, frequencies and percentages were used to evaluate and present adverse events. The total number of adverse events reported during the study period, in either the control or exercise phase, was used as the measure of safety. Adherence was assessed by calculating the attendance rate of exercise sessions completed by the total possible sessions (number of participants in the exercise condition × 18 sessions).

To address the secondary aim of the presence of elevated TNF-α levels in this cohort, the baseline measure captured at T1 was compared to previously published normative TNF-α levels [56]. The baseline measures are presented as mean and 95% confidence intervals.

For the third aim, a repeated measures ANOVA was used to determine the differences between the measured TNF-α values by within-subject factors of time (pre and post) and condition (exercise intervention and control). This method of analysis was also used to investigate the differences between secondary outcomes of inflammatory markers, muscle strength, cardiovascular fitness and body composition. The differences between the changes in TNF-α and the secondary outcomes are represented as mean and 95% confidence intervals. As part of this analysis, the between-subjects effect of condition sequence on the relative condition response was tested as a time by sequence by condition interaction term; this follows the recommendations outlined in CONSORT 2010: extension to randomised crossover trials for the testing of carry over effect [57]. Carryover was considered absent with a *p* > 0.05 [58]. A paired samples *t*-test was used post hoc to compare the difference between pre- and post-testing scores for each group.

Metabolic flexibility was only assessed before and after the exercise intervention to reduce the time-burden on the participants–with the assessments lasting ~3 h when metabolic flexibility was included in the testing battery. In assessing the effectiveness of the 6-week exercise intervention on metabolic flexibility, a two-tailed paired *t*-test was used to compare the mean within-session baseline, maximum and rate of change for RER as assessed at pre- and post-intervention. The differences between the pre- and post-intervention values for the RER outcomes are expressed as mean and 95% confidence intervals. The data used for this analysis is available as File S1: Raw Dataset.

## 3. Results

### 3.1. Participant Characteristics

**Figure 2:**
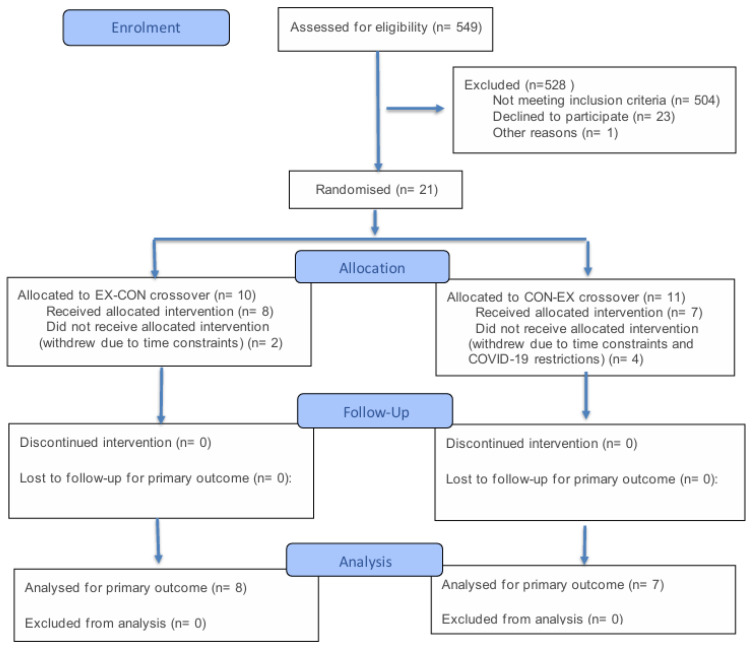
CONSORT 2025 Flow Diagram: Flow diagram of the progress through the phases of a randomised trial of two groups [that is, enrolment, intervention, follow-up and data analysis]. Note: EX-CON = exercise first then control, CON-EX = control first then exercise.

During the study recruitment period (September 2020–March 2021), 549 outpatients were screened for eligibility to participate in the study. Patient screening, contact and recruitment is described in Figure 2. A total of 21 participants were initially recruited to the study, four withdrew due to the COVID-19 restrictions in place at the hospital at which the study was conducted, while two participants withdrew due to the time constraints of the study. As randomisation occurred at the time of recruitment, there was no data collected for the six participants that withdrew. Of the participants that completed the study, eight were randomised into the EX-CON arm and seven were randomised to the CON-EX arm. Participant characteristics are shown in Table 1.

### 3.2. Adverse Events and Adherence to Intervention

There were no adverse events in response to the exercise intervention during this study. One participant fainted during the blood draw of their initial session. They were reviewed by a doctor from the State Adult Burns Unit before study participation continued.

Of the 15 participants who took part in the exercise intervention, eight attended all 18 exercise sessions, with the remaining seven participants attending at least 12 sessions throughout the 6-week study period. The overall completion rate for the prescribed exercise sessions was 90.4% (244/270 sessions). Only two participants opted to complete the sessions remotely, and those two participants reported completion of 100% of their allocated sessions (36/36).

### 3.3. Is Inflammation Elevated > 1 Year Post Non-Severe Burn?

TNF-α showed no elevation at baseline (T1) compared to previously published normative data—burned cohort (mean: 2.48 pg·mL^−1^, 95% CI: 1.49 to 3.46 pg·mL^−1^) vs. normative (mean: 5.9 pg·mL^−1^, 95% CI: 5.0 to 7.1 pg·mL^−1^) [56]. IL-6 also showed no elevation at baseline compared to previously published normative data—burned cohort (mean: 0.49 pg·mL^−1^, 95% CI: 0 to 1.65 pg·mL^−1^) vs. normative (5.19 pg·mL^−1^, 95% CI: 4.63 to 5.74 pg·mL^−1^) [59].

### 3.4. Effect of Exercise Intervention

The assumption tests for the repeated measures ANOVA showed that all residuals except for IL-2 and IL-6 were normally distributed (Shapiro–Wilk test—*p* > 0.05), and Mauchly’s test of sphericity indicated that sphericity had not been violated for any measures (*p* > 0.05). Given that all measures met sphericity, all measures were included in the repeated measures analysis.

The condition sequence had no significant effect on the differences for the primary outcome of TNF-α for exercise compared to no exercise, indicating that crossover effect was absent (*p* = 0.903). The repeated measures ANOVA showed that there were no statistically significant differences in the primary outcome of TNF-α for main effects of time or condition, or for the interaction effect (Table 2). It should be noted that a post hoc power analysis, using the change scores for this study (control: −0.53 (±1.31) pg·mL^−1^, and exercise: −0.04 (±1.08) pg·mL^−1^), and considering α < 0.05 and β = 80%, indicated that 224 participants (112 in each arm) would be required to identify a significant change between groups.

**Figure 3:**
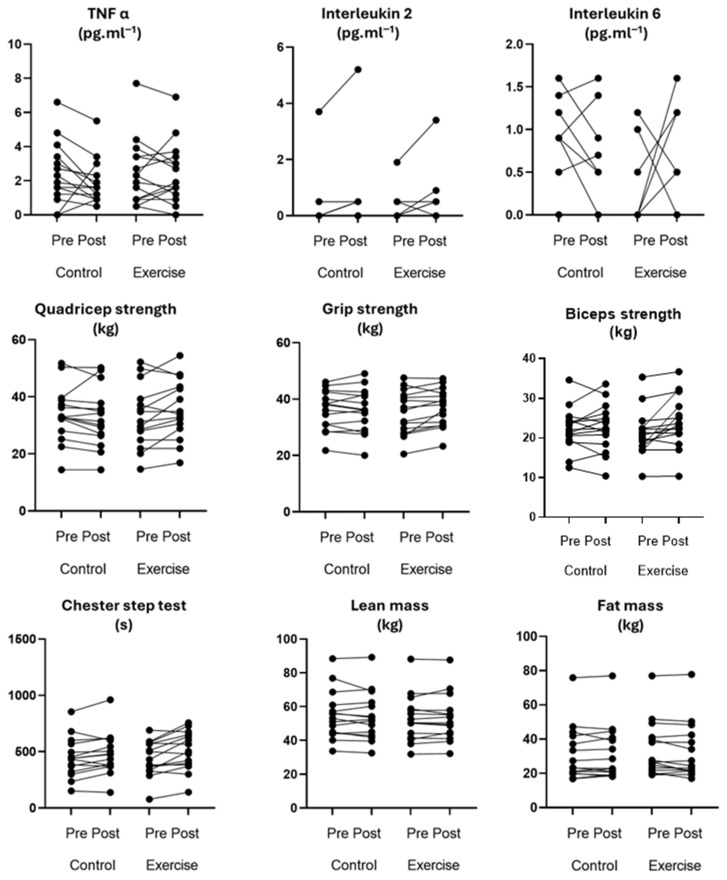
Spaghetti plots of outcome measures by condition sequence for 15 non-severely burned participants. Note: TNF = tumour necrosis factor.

For the secondary outcomes, the main effects of time for fat mass showed a significant positive association, indicating the values of the intervention and the control condition were significantly higher post intervention (Table 2). Conversely, the main effect of time for fat free mass showed a significant negative association, indicating the values of the intervention and control condition were significantly lower after intervention. The main effects of condition resulted in a significant improvement for the modified Chester step test, meaning before and after the intervention the scores were significantly improved between the intervention and control groups (Table 2). The interaction effects of condition *×* time for isometric quadriceps strength between the intervention and the control condition changed with time, and the variation ranges were different (Table 2). The between-group differences are illustrated by condition sequence in Figure 3.

A positive increase in quadriceps strength of 3.59 kg, 95% CI: 1.09 to 6.08 kg, *p* = 0.008 was observed. No other measures were statistically significant (Table 3).

No significant between-group difference was detected for baseline, maximum or rate of change in RER (Table 4). Hedge’s g indicated a small effect size for all outcomes, suggesting limited practical application.

## 4. Discussion

Our findings indicate 6 weeks of resistance and cardiovascular exercise was well adhered to (90.4% adherence rate), safe (no reported adverse events), and beneficial (quadriceps strength-mean difference: +3.59 kg, 95% CI: 1.09 to 6.08 kg, *p* = 0.008) for patients who sustained a non-severe burn > 1 year ago. Unexpectedly however, the former patients who had sustained a non-severe burn recruited in the study (>1 years post-burn) did not present with increased levels of inflammation, nor is it significantly reduced by a 6-week exercise intervention.

Rehabilitative exercise after a burn injury can improve lean body mass, physical function, clinical and physiological outcomes [10,12,16,60]. Despite the proven safety and benefit to patients [16], it is not commonly incorporated into outpatient rehabilitation after burn injury. This is also despite reviews and meta-analyses suggesting long-term implementation of exercise for post-burn recovery [10,12,14,60]. Our study indicated that 6 weeks of structured exercise for patients who sustained a non-severe burn > 1 year prior led to a 25.5% improvement in cardiovascular fitness (*p* < 0.001) and a 9% increase in quadriceps strength [*p* = 0.010] (Table 2 and Table 3). A post hoc paired samples *t*-test indicated that quadriceps strength (mean difference: +3.59 kg, *p* = 0.008) improved significantly due to the intervention. Our findings are similar to those observed in acute adult post-burn studies implementing structured exercise. These studies have reported improvements in cardiovascular fitness, muscle strength and body composition [10]. Similar changes have been observed in healthy population studies for cardiovascular fitness (11% increase) and quadriceps (12–17%) across a similar 6-week training period [61,62]. This suggests structured exercise is beneficial for adult patients who sustained a non-severe burn > 1 year ago and can be considered for prescription to this cohort as a part of a long-term management strategy.

Our findings indicated no elevation in baseline inflammatory marker levels (TNF-α and IL-6 specifically) and no evidence of changes induced due to the exercise intervention in this cohort of patients who sustained a non-severe burn > 1 year ago (Table 2 and Table 3). This differs from previous research that has indicated that after sustaining a burn injury, inflammation can remain elevated for at least 3 years post-injury [3,6]. A recent study investigated the sustained immune dysfunction in 36 paediatric patients who had sustained a non-severe burn and found that TNF-α (1.3-fold higher, *p* < 0.01) and interleukins 2 (1.2-fold higher, *p* < 0.05) and 7 (1.6-fold higher, *p* < 0.01) were significantly elevated >3 years after injury compared to a non-burned age- and sex-matched cohort [6]. Another study examining the persistence of abnormalities of various clinical parameters to assess the degree of hypermetabolic and inflammatory alterations in children that had sustained a non-severe burn found that IL-6 was significantly elevated (up to 2000-fold higher, *p* < 0.05) for up to 1100 days post-burn [3]. The marker adopted as the primary outcome in this study was based on previous work by Johnson et al. [6]; however, the marker has not been well studied in adults who have sustained a non-severe burn. The absence of elevated inflammation observed may therefore be subject to the specificity and sensitivity of the marker assessed. It is possible that non-significant findings represent a type II error, and thus a larger sample is recommended for future studies—using our own data, with an α < 0.05 and β = 80% determining a required 224 participants. Future work should examine additional markers representing the chronic inflammatory process post-burn in this cohort to better characterise burn-related inflammatory changes.

With regard to the analysis of the secondary outcome of glucose metabolism, our findings indicated there was no evidence of change due to exercise in baseline, maximum or rate of change in RER (Table 4). Prior to this study, metabolic flexibility has not been investigated in the burned population, with previous metabolic research focusing on resting energy expenditure as a measure of increased energy demand, without investigating any fluctuation in macronutrient composition in the post-burn cohort [28,63]. There is limited evidence of prolonged exercise-training responses in other populations with regard to metabolic flexibility; however, it is well documented that acute exercise promotes mechanistic epigenomic and proteomic changes in skeletal muscle that lead to greater energy production and improved metabolic flexibility [64]. Future research into the effects of exercise on post-burn injury metabolism should focus on mechanistic changes in the skeletal muscle that will promote improved metabolic flexibility.

The strength of our study includes its novelty in assessing the effectiveness of exercise in altering the physiological response of non-severely burned adult patients who sustained their injury > 1 year ago; demonstration of the safety and exercise-related benefits of structured exercise in this cohort; and the inclusion of non-severely burned patients.

This study has several limitations that should be acknowledged. The primary limitation is small sample size, with 21 participants recruited and only 15 completing the crossover design. This limited sample size reduces the statistical power of the findings and restricts the generalisability of the results to the broader burns population. Additionally, blinding of the researchers to group allocation was not possible, which may have introduced assessor bias. The collection of metabolic flexibility, strength and cardiovascular measures by a non-blinded assessor further increased the potential of bias in outcome assessment; however, standardised randomisation and assessment protocols were used to mitigate this where possible. Furthermore, the potential for residual exercise effects in participants who completed the exercise arm first may have influenced their subsequent control period, potentially limiting the ability to fully isolate the independent effects of each condition within the crossover design.

## 5. Conclusions

Rehabilitative exercise appears to be safe, feasible and beneficial for non-severely patients who sustained their injury > 1 year ago. However, our study showed no presence of burn-induced changes to the inflammatory markers or glucose metabolism, which precluded our ability to determine their response to exercise. Importantly, the study was not adequately powered to detect significant changes in TNF-alpha, and therefore these findings should be interpreted with caution.

## Figures and Tables

**Table 1:** Descriptive characteristics of 15 non-severely burned participants presented as combined total and crossover group designation (EX-CON or CON-EX). Continuous variables presented as mean (standard deviation), and categorical variables presented as frequency and percentage. Normal HbA1c defined as ≤6.5%. HbA1c was only available for 11 participants in the study (EX-CON = 5, CON-EX = 6).

	Burn Survivors [n = 15]	EX-CON [n = 8]	CON-EX [n = 7]
Age [y]	42 [15.9]	43 [17.3]	40 [15.4]
Height [m]	1.65 [0.1]	1.65 [0.1]	1.64 [0.1]
Body mass [kg]	86.7 [25.7]	85.4 [27.0]	88.2 [26.2]
Total burned surface area burned [%]	9.6 [4.6]	7.7 [3.3]	11.7 [5.2]
Average sessions attended [/18]	16 [2.3]	17 [1.9]	16 [2.8]
%1RM per session	75 [2.3]	76 [0.9]	74 [3.1]
HbA1c % at baseline	Normal—10 [90.9%] Above normal—1 [9.1%]	Normal—5 [100%]	Normal—5 [83%] Above normal 1 [17%]

Note: 1RM = 1 repetition maximum, HbA1c = glycated haemoglobin. EX-CON = exercise first then control, CON-EX = control first then exercise.

**Table 2:** Comparison of outcome measures between the two conditions before and after the intervention. Presented as mean ± standard deviation. * denotes a significant *p* value.

Measurement	Control			Exercise			Time		Condition		Condition × Time	
	Pre	Post	Change	Pre	Post	Change	F	P	F	P	F	P
Primary Outcome: TNF-alpha [pg·mL^−1^]	2.45 ± 1.78	1.92 ± 1.28	−0.53 ± 1.31	2.49 ± 1.91	2.45 ± 1.78	−0.04 ± 1.08	0.971	0.342	1.50	0.242	1.356	0.265
Secondary Outcomes: Interleukin-2 [pg·mL^−1^]	0.31 ± 0.95	0.55 ± 1.31	0.23 ± 0.42	0.23 ± 0.51	0.23 ± 0.29	0.17 ± 0.46	0.727	0.409	3.421	0.087	0.430	0.523
Interleukin-6 [pg·mL^−1^]	0.49 ± 0.59	0.37 ± 0.55	−0.12 ± 0.38	0.18 ± 0.39	0.33 ± 0.55	0.15 ± 0.65	2.204	0.162	0.156	0.700	1.804	0.202
Grip strength [kg]	36.12 ± 6.91	35.9 ± 7.69	−0.22 ± 1.92	35.07 ± 7.82	36.9 ± 6.77	1.83 ± 2.68	0.002	0.967	4.466	0.054	4.239	0.060
Quadriceps strength [kg]	33.91 ± 9.64	32.75 ± 10.34	−1.16 ± 3.53	32.97 ± 10.91	35.4 ± 10.39	2.43 ± 4.40	1.113	0.311	0.647	0.435	9.046	0.010 *
Biceps strength [kg]	22.42 ± 5.16	22.62 ± 6.04	0.20 ± 3.02	21.99 ± 5.42	23.27 ± 4.65	1.28 ± 4.61	0.092	0.766	0.972	0.342	0.485	0.498
Modified Chester Step Test [s]	447.3 ± 177.8	479.8 ± 185.2	32.47 ± 52.52	439.7 ± 155.9	507.6 ± 173.6	67.93 ± 74.46	0.673	0.427	25.356	0.001 *	1.661	0.220
Fat mass [kg]	31.33 ± 15.98	31.51 ± 16.10	0.18 ± 2.23	33.79 ± 16.04	32.59 ± 16.53	−1.19 ± 2.53	9.564	0.009 *	2.413	0.144	1.775	0.206
Fat free mass [kg]	54.92 ± 14.37	54.27 ± 14.39	−0.65 ± 2.86	52.89 ± 13.96	53.22 ± 13.91	0.34 ± 2.08	15.399	0.002 *	0.084	0.776	1.049	0.325

Note: TNF = tumour necrosis factor.

**Table 3:** Paired samples *t*-test intragroup comparison for study measures. * denotes significant *p* value.

Measurements Exercise-Control	Mean Difference ± SD	95% CI	*p* Value
TNF-α [pg·mL^−1^]	+0.50 ± 1.61	−0.39 to 1.39	0.249
Interleukin-2 [pg·mL^−1^]	−0.60 ± 0.32	−0.24 to 0.12	0.483
Interleukin-6 [pg·mL^−1^]	+0.27 ± 0.76	−0.15 to 0.69	0.187
Grip strength [kg]	+2.05 ± 3.70	0.00 to 4.10	0.050
Quadriceps [kg]	+3.59 ± 4.50	1.09 to 6.08	0.008 *
Biceps [kg]	+1.08 ± 5.56	−2.00 to 4.16	0.466
Modified Chester Step Test [s]	+35.47 ± 100.93	−20.4 to 91.4	0.195
Fat mass [kg]	−1.37 ± 3.81	−3.48 to 0.74	0.185
Fat free mass [kg]	+0.99 ± 3.53	−0.96 to 2.95	0.294

Note: TNF = tumour necrosis factor, SD = standard deviation, CI = confidence interval.

**Table 4:** Pre- and post-exercise intervention metabolic flexibility outcome measures presented as mean [standard deviation] and post-pre difference (95% confidence intervals).

Measurement	Exercise				
	Pre	Post	Post-Pre (95% CI)	Hedge’s g	*p* Value
Baseline RER	0.85 (0.053)	0.86 (0.069)	0.01 (−0.02 to 0.05)	0.16	0.561
Maximum RER	0.94 (0.048)	0.95 (0.057)	0.01 (−0.01 to 0.04)	0.19	0.579
Rate of change [per h]	0.156 (0.118)	0.136 (0.075)	−0.02 (−0.08 to 0.04)	0.20	0.624

Note: RER = respiratory exchange ratio, CI = confidence interval.

## Data Availability

The original contributions presented in this study are included in the article/Supplementary Material. Further inquiries can be directed to the corresponding author.

## Supplementary Materials

The following supporting information can be downloaded at https://www.mdpi.com/article/10.3390/ebj7010011/s1, File S1: Raw dataset.
